# The Role of Emotional Information in Banner Blindness

**DOI:** 10.3389/fpsyg.2022.813440

**Published:** 2022-05-10

**Authors:** Frol Sapronov, Elena Gorbunova

**Affiliations:** Laboratory for Cognitive Psychology of Digital Interfaces USER, HSE University, Moscow, Russia

**Keywords:** banner blindness, visual attention, emotions, valence, arousal, usability

## Abstract

Modern theories in affective science postulate that emotional stimuli can affect subject’s attention. Emotional stimuli can guide and capture visual attention, which may be related to evolutional importance of quick reactions for emotional objects in the real life. The study examined the influence of valence and arousal of advertisement on the banner blindness phenomenon—ignoring the advertisement or interface details similar to it on the website. In two experiments participants were asked to find the information on the website, where different banners were placed. In the first experiment banners had the same valence, but different arousal. In the second experiment, the banners had different valence, but equal arousal. Contrary to the classical studies in affective science, we found that banners with neutral valence were recognized better as compared to negative and positive ones. The results are discussed in terms of user experience contributing to banner blindness occurrence.

## Introduction

The impact of digital interfaces on cognitive processes has increased as gadgets quickly entered daily human use (e.g., Levy et al., 2016; Wilmer et al., 2017). Almost all commercial, educational, and other public institutions have their own websites where users can find any information. Technology development and the use of Internet have significantly facilitated learning, work, and communication. This shift has been characterized by an increasing need to process huge amounts of information, both relevant and irrelevant (Heylighen et al., 2004). As individuals are confronted with the large amount of information in their daily life, cognitive processes adapt to avoid information overload (Kirsh, 2000). One of the main regulators is attention. Irrelevant information does not draw enough attention and as a result is ignored, distorted, or forgotten. The same trend can be observed in the human-computer interaction. The lack of clear separation between relevant and irrelevant information can seriously reduce the user’s efficiency. In an environment full of overwhelming stimuli, it can be extremely difficult to focus on the current task. In addition, information overload and various distractions, such as messages, pop-up advertisements, and other notifications increase the user’s cognitive load, which, in turn, hinders the successful achievement of a current goal (Roda, 2011). In particular, noticeable elements automatically capture user’s attention: for example, on a static display, attention is drawn to moving objects (Theeuwes, 2010). Moreover, some authors argue that distractors such as annoying ads can reduce user’s subjective satisfaction and lead to site abandonment (Goldstein et al., 2015). The advertisement’s presence is also linked to less user satisfaction with search results and higher level of frustration while using the website (Foulds et al., 2021).

Despite the popularity of banner advertising, it is not always effective. The phenomenon of “banner blindness” that this article addresses is named among the reasons. It means that users may not notice advertising banners and interface elements similar to them. In addition to “blindness” for banners, poorly positioned or poorly drawn site elements that resemble banner ads can also be ignored. When looking into the phenomenon of “banner blindness,” the results can be used to create a noticeable type of advertising (Çiçek et al., 2017), as well as to create “smart” interfaces (Roda, 2011). In the study of Hsieh et al. (2012), several possible mechanisms for the occurrence of banner blindness were examined in detail: information type, user experience, habituation, and attention inertia.

Attention can be divided into endogenous and exogenous. Endogenous attention is controlled by humans and can be attributed to top-down processing. Exogenous attention is uncontrollable and depends on an external stimulus (Chun and Wolfe, 2001). Thus, when the user is distracted by the screen brightness change, popup windows, and other stimuli, it can be attributed to the exogenous attention. The main type of information that the user encounters during interaction with the interface is textual information. Since the text analysis itself is carried out by complex and resource-intensive cognitive mechanisms, it requires endogenous attention. When searching for the necessary information on the website users focus on the textual information. The analysis of banners does not take place at the same level, so they are not noticed. Probability of detecting the banner increases if the user does not have a clearly defined goal to read the text, which is supported by studies of eye movements (Tang and Jhuang, 2005). Similar results have been found in more recent studies with eye tracking: the ads attracted more attention during free browsing than during the reading task (Simola et al., 2011).

Banner blindness depends not only on the level of information processing but also on the previous user experience (Yu and Tao, 2009). For example, the presence of ads always affects task performance, which means that users are aware of their presence and deliberately ignore them (Pasqualotti and Baccino, 2014). Banner ads can be quickly assessed as unnecessary and repetitive information and therefore skipped by the user. Also, banners are usually located in the same areas of the websites, which further allows individuals to transfer the previous experience of banner perception to different sites. It is shown that users are more likely to interact with interface’s details in the vertical zones (Sulikowski and Sulikowski, 2021). An explanation of this mechanism can be found in modified theories of early attentional selection, in which irrelevant stimuli move to the second stage of analysis but are analyzed only if they have particular importance (Treisman, 1969). Within the framework of these theories, we can also talk about the impact of interference effects on the current task and the interaction of top-down and bottom-up processes: user experience affects the interaction with the environment. Interference effects can be considered as delays in the processing of target stimuli due to distractors. For example, if distractors are contextually similar to the current task, or have some unique features, they can distract the user’s attention from target processing. The amount of interference caused by distractors is also likely to be related to the previous user experience. Therefore, one of the ways to overcome banner blindness is to make the content of banner advertising task-relevant according to the content of the site (Wojdynski and Bang, 2016). According to the eye-tracker study, the quality of ads may affect the amount of visual attention deployed to banners: participants pay more attention to high quality advertisement (Buscher et al., 2010).

More recent research revealed that habituation and attention inertia also affect the banner blindness. Habituation is adaptive desensitization to familiar stimuli due to their irrelevance. Attention inertia is the tendency to focus on a specific and relevant object. Anderson et al. (1987) found that the effect of distractors was most negligible in the middle of a task. But at the beginning and the end of the task their influences increases (Anderson et al., 1987). This means that the banner which acts as a distractor on the website will be more effectively detected and remembered by the user at the beginning and at the end of the interaction with the website. User sensitivity to banner ads also increases at the beginning and at the end of the task of searching for the information (Day et al., 2006). Based on research results, marketers try to make ads more visible—for example, they can use pop-up notifications to attract involuntary attention, or try to make ads relevant to user tasks. One of the ways to manipulate the user’s attention is to create emotional ads.

Many studies show that there is some connection between emotions and cognitive processes. Emotion–induced blindness is one of the phenomena studied within this problem area (Divita and Meera, 2017). If two stimuli—emotional and neutral—are presented one after each other in a short time succession, emotional stimuli usually «win» the processing competition and are memorized better (e.g., Most and Wang, 2011; Kennedy and Most, 2012; Wang et al., 2012).

Talking about the emotional characteristics of stimuli, we first of all mean valency—the degree of positive or stimulating affective reaction that the image causes and arousal—the intensity of the affective reaction that the image evokes (Kurdi et al., 2017). It is assumed that stimuli with higher arousal have a processing advantage over stimuli with lower arousal. There is no certain opinion regarding the role of valency in the attentional and perceptual processing. However, it was revealed that both unpleasant and pleasant images capture attention more as compared to the neutral images (Nummenmaa et al., 2006). Several studies revealed that scary, disgusting, and erotic stimuli are processed better (e.g., Lang et al., 2008). Emotional stimulus captures the subject’s attention, which in turn allows this element to move to a later stage of processing (Beanland et al., 2018).

However, emotional stimuli do not always attract attention. It has also been revealed in a number of studies (e.g., Pessoa, 2005; Vuilleumier, 2005). For example, participants ignored distracting emotional stimuli, while they were solving difficult experiment tasks.

In this study, we want to reveal the influence of affective information on the banner blindness occurrence, which is a new and little-studied area. Research in this research area can provide new insights into the existence of a causal relationship between affective and cognitive processes, which, in turn, opens up new fields for research in both psychology and marketing. For example, we hope that our study will add new data on the emotional factors influencing the occurrence of banner blindness. Also, those results could be implemented in marketing to make the advertising more noticeable for users. Research aimed at examining the influence of emotional characteristics of stimuli on memorization can provide an understanding of the interaction of these processes. It will lead to a more confident conclusion about the nature of such phenomena as blindness caused by emotions and attention motivated by emotions. Also, such studies can complement the already existing knowledge about user’s behavior: the impact of experience on working with digital interfaces, the phenomena of “banner blindness” and inattentional blindness.

In two experiments, we manipulated emotional features of the presented banners—arousal (Experiment 1) and valence (Experiment 2). According to the studies in emotional information processing, emotional stimuli are suggested to capture user’s attention, which is expected to lead to better recognition of the banners with high arousal, as well as for banners with positive or negative valency compared to neutral ones. On the contrary, as banners are usually assumed to be emotional, and tend to be ignored by users, banner blindness also can be induced more by emotional stimuli as compared to neutral ones.

## Experiment 1

In the first experiment, we estimated how does the arousal of images, used as the banners, influences the probability of banner blindness to appear.

### Method

#### Participants

Eighty volunteers took part in the experiment (22 male, 58 female, aged 18–29 years, and *M* = 20.3). The participants were recruited through social networks. All participants, or their legal guardians, gave the informed consent to pass the experiment and data processing. All participants were native Russian speakers, naive to experimental hypotheses, and had no neurological disabilities.

#### Stimuli

To select stimuli the pilot study was carried out. In accordance with the dimensional model of emotions, we decided to investigate the influence of valence and arousal separately (Faith and Thayer, 2001). Images were chosen from the OASIS photograph database (Kurdi et al., 2017). This database collected 900 images found on the Internet, after which their emotional characteristics were determined on a large American sample. We sorted and chose some images according to their arousal and valency values in R software development. The first part of our pilot study was carried out to check the reproducibility of data on the Russian sample and to improve the quality of the selection of images. Participants (*N* = 15, did not participate in the main experiment) were presented with images from the original database, which were chosen earlier. Participants had to evaluate on a scale from 1 to 7 in terms of valence and arousal, as in the original database article. The data collected in this part of pilot study appeared to be representative. Also, based on the results of the pilot study, the most suitable photographs were selected.

**Figure 1:**
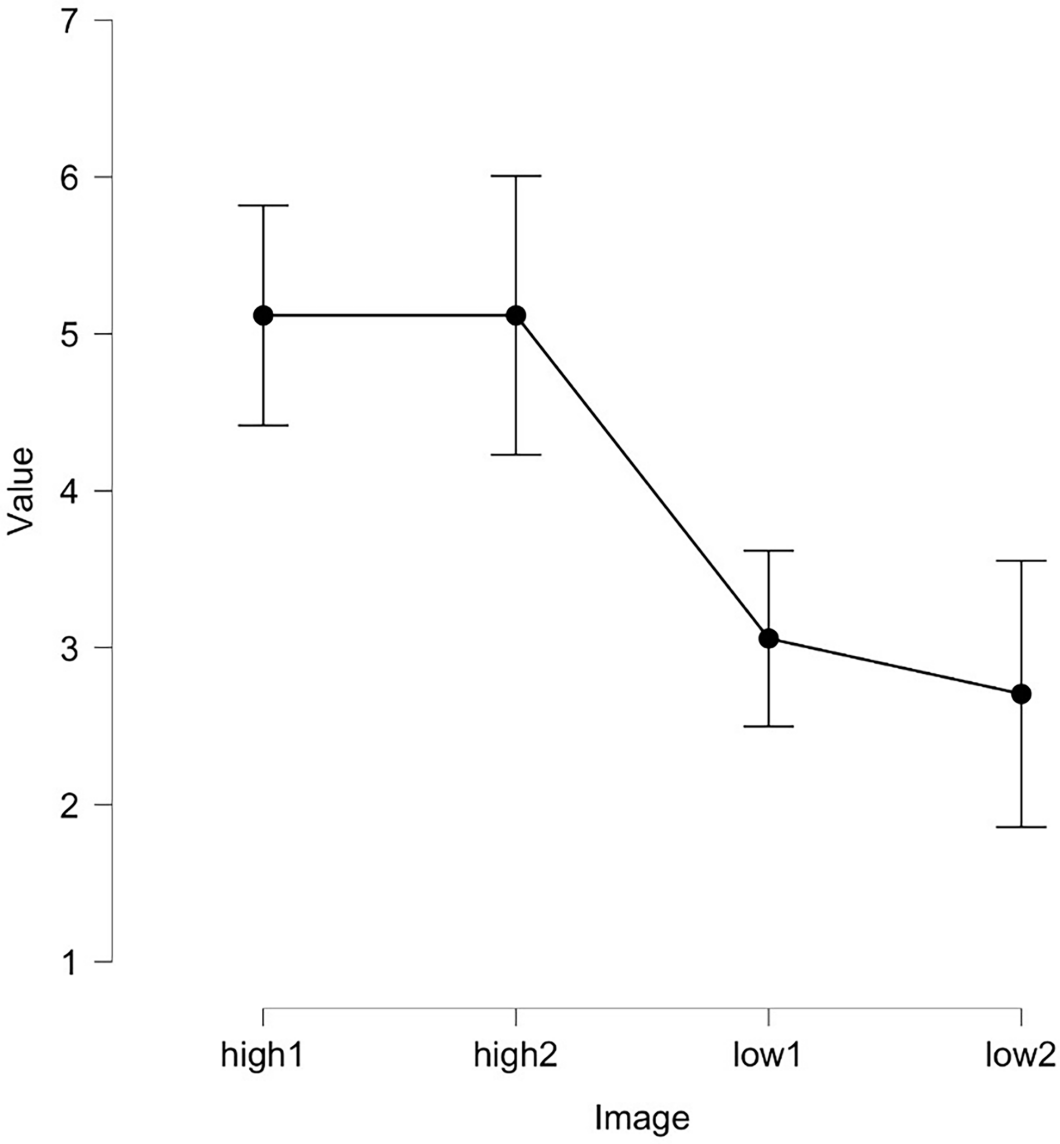
Arousal values of images from experiment 1.

After the first part of pilot study, selected images were equalized in terms of brightness. In the second pilot study, participants (*N* = 13, did not participate in the first part of the pilot study and in the main experiment) also estimated the arousal and valence of the images that were selected after the first part of pilot study and subsequently processed. The data collected in the second pilot study also appeared to be representative. We compared the scores of the pilot subjects using repeated measures ANOVA, banners were statistically significantly different from each other in terms of arousal values *F* (3, 64) = 13.1, *p* < 0.01 (see Table 1; Figure 1). Images were placed in locations typical for banners: above and on the side of the main information. We intentionally did not add titles or frames to the images (despite it is commonly used in advertising), because that could affect the emotional features on which our experiment was focused. Now, we can safely claim that banner blindness does not only apply to advertisement, but also to the details of interface, that are in some way similar.

**Table 1:** *Post-hoc* comparisons of arousal values (pilot data).

		*SE*	*t*	*p* (Tukey correction)
High1	High2	0.5	<0.001	>0.999
	Low1	0.5	4.05	<0.01
	Low2	0.5	4.75	<0.01
High2	Low1	0.5	4.05	<0.01
	Low2	0.5	4.75	<0.01
Low1	Low2	0.5	0.69	0.89

#### Procedure

The participants were registered through a survey in Google Forms, after which they were individually coded and randomly assigned to one of two groups: A1—high arousal of banners, A2—low arousal of banners.

The experiment was conducted online. The respondents were sent the general information about the experiment (duration, general purpose, and procedure) and instruction. After reading the instruction, participants were sent a task and an experiment file.

**Figure 2:**
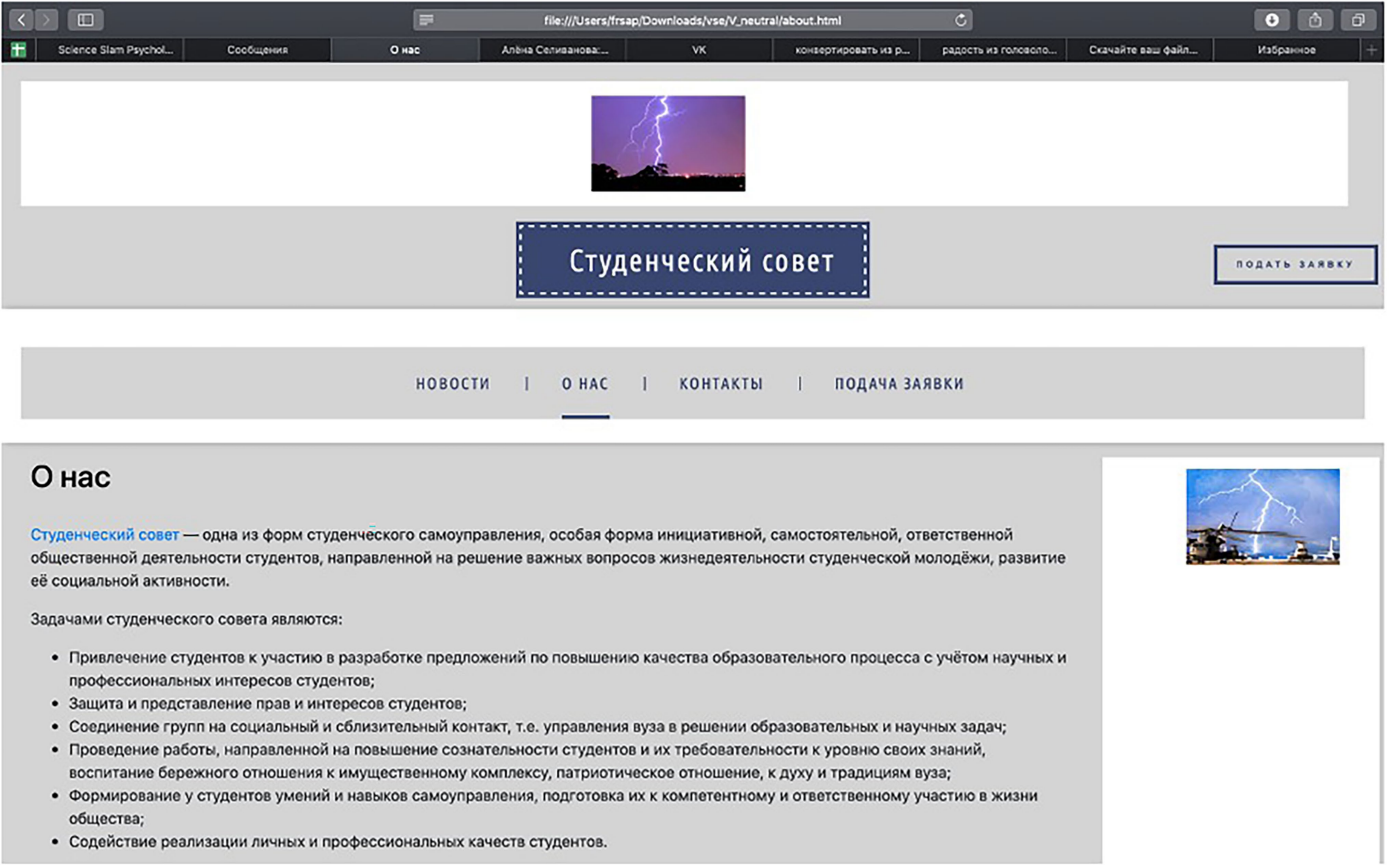
Example of the website.

The websites were created specially for the experiment and looked like a real student council website. They contained information about the main goals, available positions, and opportunities to become a team member. Depending on the group, the participants used a link to one of two possible websites. Websites were equal except the type of banners that we used: in one condition, banners had high arousal, in other condition, they had low arousal. Each website had two different banners (both with high arousal or low arousal): above and on the side of the main information (see Figure 2 for an example). The participants were instructed to find a link that would allow them to apply for participation in the student council. To do this, the participants had to familiarize themselves with the content of the web site and find a link to a Google Form, which they subsequently filled out.

There were questions in the Google form, what the participants filled at the end of experiment, which were further analyzed by us:

Have you seen any pictures (banners)? The possible answer could be “yes” or “no.” To reveal if the participants saw the banners, and not something else, we subsequently asked them to briefly describe the appearance of the banners.

**Figure 3:**
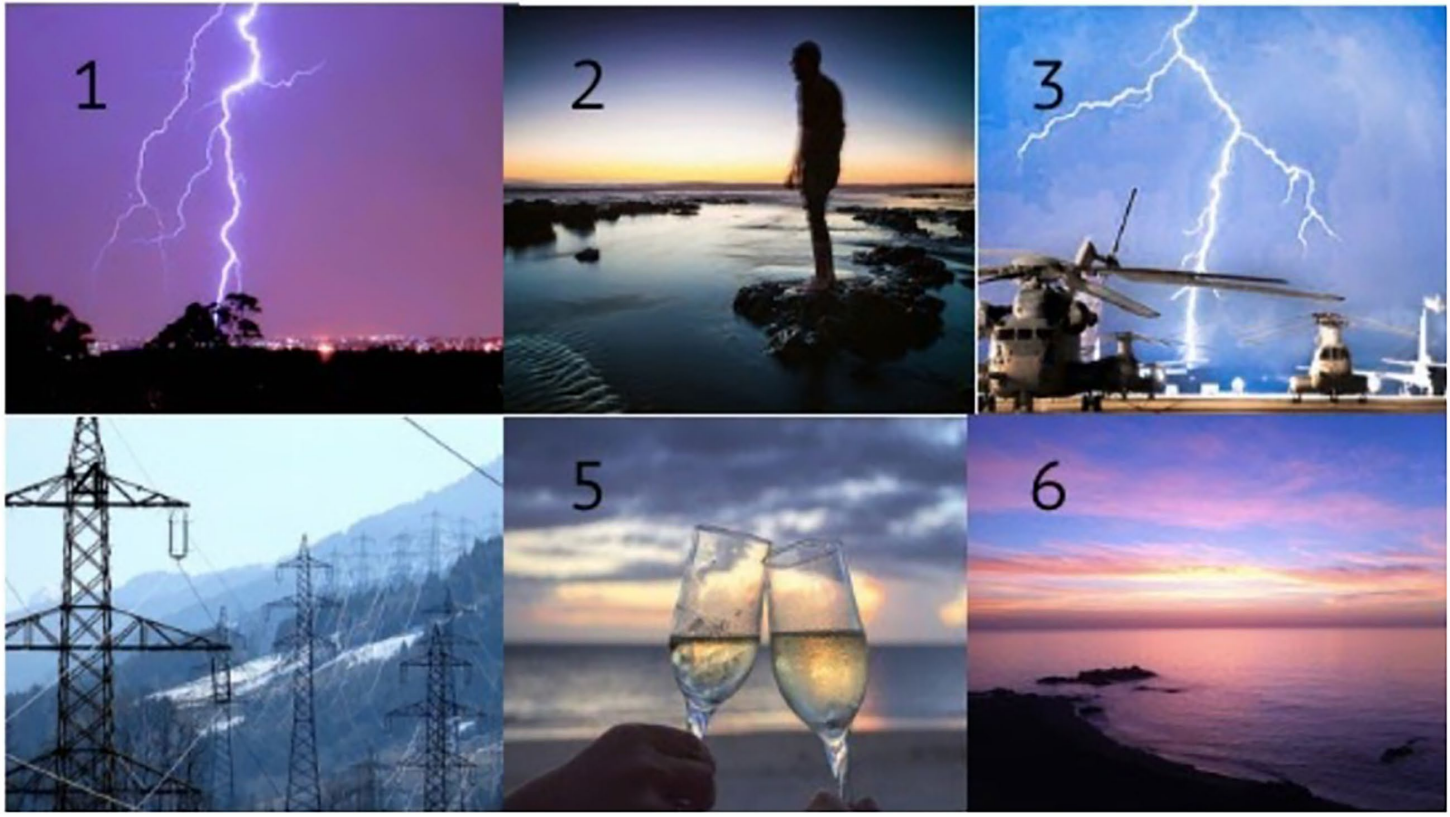
Banner stimuli presented for recognition (Marsden et al., 2018). Images available at https://www.oasis-database.org.

Have you seen any of these banners? (see Figure 3, for an example). Participants should indicate the banners that they saw from the list of six banners. If participants did not recognize any banners, they were proposed to guess.

**Figure 4:**
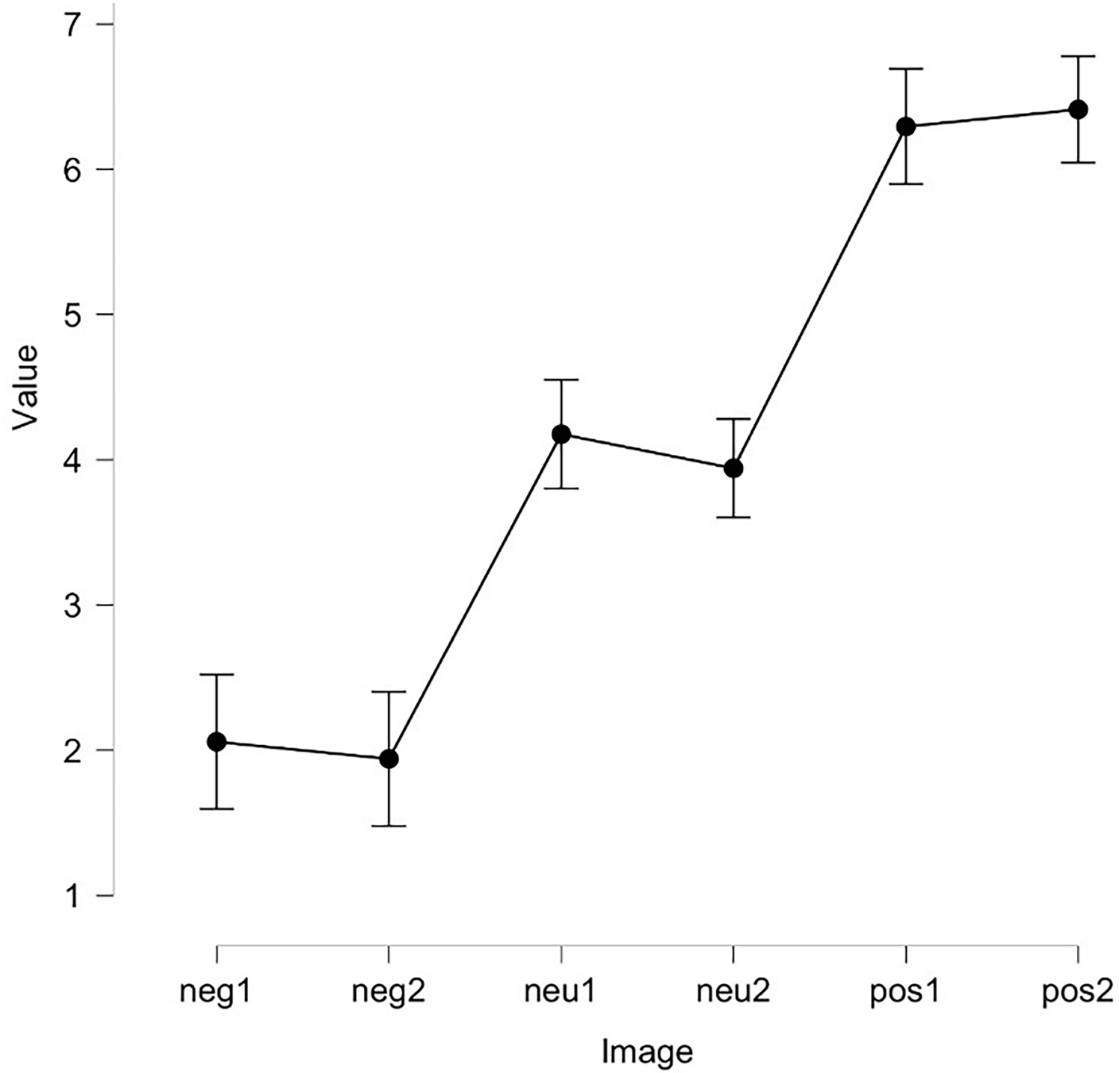
Valence values of images from Experiment 2.

### Results

Data analysis was performed in the R software environment (version 3.6). All participants successfully found the link and answered the questions. The first question was to report about the presence banners on the website. Responses “did not see banners” were assigned 0, responses “saw banners” were assigned 1. Based on these responses, a contingency table was created (see Table 2).

**Table 2:** Summary table of found and missed banners with high and low arousal (cells contain the number of participants).

	High arousal	Low arousal
Saw banners	28	29
Did not see banners	12	11

**Table 3:** Summary table of found lateral and top (cells contain the number of participants).

	High arousal	Low arousal
To the right of the text region	8	4
Above of the text region	10	12

The analysis of memorizing the presence of stimuli was carried out using the Pearson Chi-square. The constructed model turned out to be statistically insignificant for memorizing the presence of stimuli, χ^2^ (1, *N* = 80) = 0.06; *p* = 0.80.

We also compared the frequency of remembering banners localized on top and on the side of the main information (see Table 3). No significant differences were found in the banner memorization depending on its location, χ^2^ (1, *N* = 34) = 1.4; *p* = 0.23. There were also some false alarms, when those participants reported about seeing a banner but made an error in the recognition task. But we did not find the significant differences in number of false alarms in this analysis as well, χ^2^ (1, *N* = 80) = 1.92; *p* = 0.16.

The second question was to recognize the banners presented on the website. Six images were shown to the participants. Two of these images were located on the website, where the experiment task was, other were used as distracters. Participants had to recognize the banners and to indicate which of them did they saw. A multinomial logistic regression was used for the analysis. Participant’s responses were coded as: “did not recognize any banners,” “recognized one banner,” and “recognized both banners,”—0, 1, and 2, respectively (see Table 4). If participants correctly recognized one of two banners, we marked this answer as 1. The arousal of stimuli was used as predictors, where high arousal was chosen as a reference group (see Table 5 for summary). Some participants did not report about seeing a banner but correctly made the recognition task, but it was insignificant χ^2^ (1, *N* = 80) = 1.01; *p* = 0.31.

**Table 4:** Summary table of recognized banners with high and low arousal.

Recognition	Arousal		Total
	High	Low	
Recognized one banner (coded as 1)	18	16	34
Recognized two banner (coded as 2)	9	12	21
Did not recognize banners (coded as 0)	13	12	25

Based on the obtained model, we can say that the influence of both high and low arousal of the stimulus on the recognition of one banner, as well as on both banners is statistically insignificant, the results are presented in Table 5. The code for analysis can be found at the Open Science Framework following link: https://osf.io/xje2u/?view_only=5203e0c32e924df3ab3694f2363d82b4.

**Table 5:** Multinomial logistic regression results, where high arousal was chosen as a reference group (Experiment 1).

Arousal	Effect on one banner recognition	Effect on two banners recognition
High	*Intercept* = 0.45, *z* = 0.07, *p* = 0.94	*Intercept* = 0.99, *z* = −0.61, *p* = 0.53

### Discussion

In this experiment, we did not find any significant differences in remembering the presence of banners with different arousal. Significant differences in recognition accuracy were not found as well. So, according to our experiment, it can be assumed that arousal of the banner does not influence its memorizing and recognition.

It is important to note that the banners had neutral valence and differed only in the level of arousal: high or low. Thus, we can say that the level of the banner’s arousal by itself does not affect the user’s attention. It seems that emotional characteristics are not read automatically but are determined only after the attention is transferred to the stimulus (e.g., Pessoa et al., 2003). This means that there are two possible interpretations of the obtained results. First, the stimuli could be missed by users at the early stages of information processing, since they looked like typical banners: bright, located to the right and left of the main information and a certain shape, and simply not analyzed by their emotional features at the lately stages of processing. Otherwise, the banners could be recognized, but arousal may have no effect on banner recognition.

It is likely that the user experience helps to recognize the information as relevant. Thus, images in a certain place of the site might be perceived as unnecessary in advance. In this regard, users ignore banners, knowing that they can distract them from the task. In our study the participants were looking for a specific link on the site, and probably spent a plenty of cognitive resources. Accordingly, the resources of attention could not be enough to view images that are known to be identified as irrelevant. This is also supported by other studies. Users who aimlessly browse a website are more likely to notice banners than users who have a purpose of staying on the site (Pagendarm and Schaumburg, 2001). If the banners were filtered out on the early stage of information processing, then users could not assess their emotionality at the later stages of processing. However, the percentage of users that did not see any banners in our study, is quite low, so the probability of filtering the banners at the early stage of processing does not seem relevant here.

On the other side, there are studies that demonstrate that banner blindness consists precisely in actively ignoring banners (Burke et al., 2004). In that case, a level of arousal should indicate that the information is irrelevant. This is consistent with Ying and colleagues findings that the more salient an ad is, the more it provokes banner blindness (Ying et al., 2009). Therefore, the increase in the phenomenon of banner blindness can be influenced not only by physical characteristics (animation, brightness, color, and location), but also by emotional characteristics. In this experiment, the level of arousal had no effect on banner recognition. However, even if the arousal does not affect banner recognition, other emotional features may be relevant for it. In the second experiment, we addressed this issue manipulating the valence of the banners.

## Experiment 2

In the second experiment, we estimated how does the valence of images, used as the banners, does influence the probability of banner blindness to appear.

### Method

#### Participants

Around 120 volunteers took part in the experiment (41 male, 79 female, aged 18–29 years, *M* = 19.74). The participants were recruited through social networks. All participants, or their legal guardians, gave the informed consent to pass the experiment and data processing. All participants were native Russian speakers, naive to experimental hypothesis, and had no neurological disabilities.

The participants were registered through a survey in Google Forms, after which they were individually coded and randomly assigned to one of three groups: V1—positive valence of banners, V2—neutral valence of banners, and V3—negative valence of banners. In this experiment, we were interested in the influence of valence on recall and recognition of banners. The participants were divided into three different groups because each participant can only do the experiment once as the phenomenon of banner blindness might not occur.

#### Stimuli

The choice of stimuli in Experiment 2 was similar to Experiment 1. The only difference was that the participants evaluated the valence of banners, and not their arousal. We compared the scores of the pilot subjects. ANOVA showed that the banners were significantly different from each other in terms of valence values: *F* (5, 96) = 105.2, *p* < 0.01 (see Table 6; Figure 4).

**Table 6:** *Post hoc* comparisons of valence values (pilot data).

		*SE*	*t*	*p* (Tukey correction)
Neg1	Neg2	0.26	0.43	0.998
	Neu1	0.26	−7.87	<0.01
	Neu2	0.26	−7.00	<0.01
	Pos1	0.26	−15.75	<0.01
	Pos2	0.26	−16.19	<0.01
Neg2	Neu1	0.26	−8.31	<0.01
	Neu2	0.26	−7.44	<0.01
	Pos1	0.26	−16.19	<0.01
	Pos2	0.26	−16.63	<0.01
Neu1	Neu2	0.26	0.87	0.96
	Pos1	0.26	−7.87	<0.01
	Pos2	0.26	−8.31	<0.01
Neu2	Pos1	0.26	−8.75	<0.01
	Pos2	0.26	−9.19	<0.01
Pos1	Pos2	0.26	−0.43	0.99

**Figure 5:**
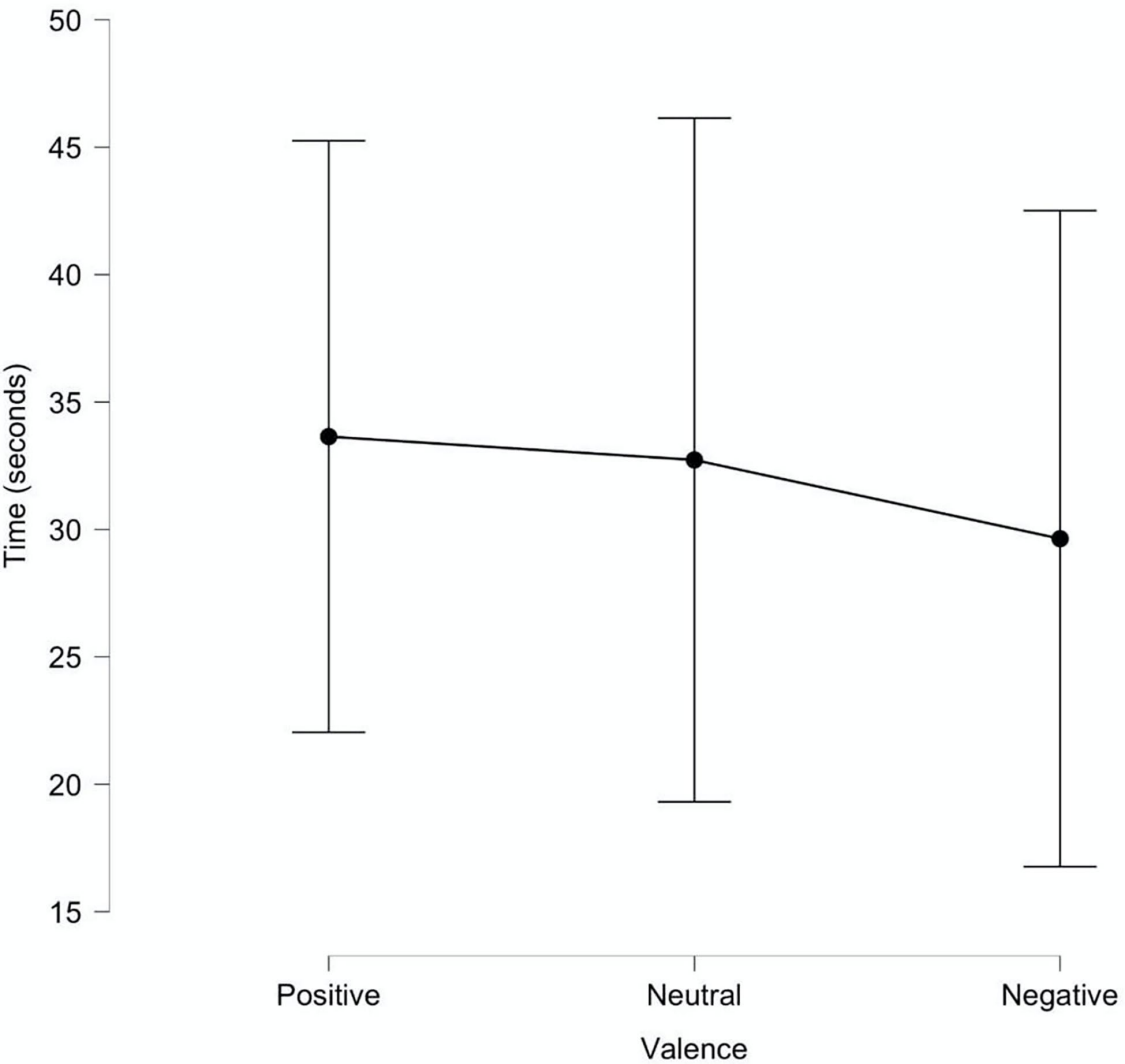
Time spent on websites.

#### Procedure

The design of Experiment 2 was similar to Experiment 1. The only differences were in the number of groups (three instead of two) and type of stimuli that we used as banners.

### Results

Data analysis was performed in the R software environment (version 3.6).

The first question in Experiment 2 was similar to Experiment 1. Responses «did not see banners» were assigned 0, responses «saw banners» was assigned 1. Based on these responses, a contingency table was created (see Table 7).

**Table 7:** Summary table of found and missed banner with positive, neutral, and negative valence.

	Positive valence	Neutral valence	Negative valence
Saw banners	31	32	29
Did not see banners	9	8	11

**Table 8:** Summary table of found lateral and top (cells contain the number of participants).

	Positive valence	Neutral valence	Negative valence
To the right of the text region	14	7	12
Above of the text region	7	13	5

The analysis of memorizing the presence of stimuli was carried out using the Pearson Chi-square. The constructed model turned out to be statistically not significant for memorizing the presence of stimuli, χ^2^ (2, *N* = 120) = 1.18; *p* = 0.55.

We also compared the frequency of remembering banners localized on top and on the side of the main information (see Table 8). Significant differences were found in banner memorization depending on its location, χ^2^ (1, *N* = 58) = 6.02; *p* = 0.049. There also were some false alarms, which mean that participants reported about seeing a banner but made an error in the recognition task. But we did not find significant differences in the number of false alarms in this analysis, χ^2^ (2, *N* = 120) = 3.27; *p* = 0.19.

The second question in Experiment 2 was also similar to Experiment 1. Multinomial logistic regression was carried out; valence of stimuli was used as predictors: positive, neutral, and negative, where neutral valence was chosen as a reference group (see Table 9 for summary). Some participants did not report about seeing a banner but correctly made the recognition task, but it was insignificant, χ^2^ (2, *N* = 120) = 1.87; *p* = 0.39.

**Table 9:** Summary table of recognized banners with positive, neutral, and negative valence.

Recognition	Valence			Total
	Positive	Neutral	Negative	
Recognized one banner (coded as 1)	21	20	17	58
Recognized two banner (coded as 2)	2	9	4	15
Did not recognize banners (coded as 0)	17	11	19	47

Based on the obtained model, we can say that the probability of recognizing the banners with neutral valence is significantly different from the probability of recognizing the banners with negative and positive valence. Since the coefficients have negative values, we can talk about an inversed relationship, which means that the probability of recognizing a negative or positive banner is lower than a probability to recognize a neutral one, the results are presented in Table 10. The code for analysis can be found at the following link https://osf.io/xje2u/?view_only=5203e0c32e924df3ab3694f2363d82b4.

**Table 10:** Multinomial logistic regression results, where Neutral valence was as a reference category (Experiment 2).

Valence	Effect on one banner recognition	Effect on two banners recognition
Positive	*Intercept* = 0.09, *z* = −1.76, *p* = 0.1	*Intercept* = 0.09, *z* = −1.28, *p* = 0.02
Negative	*Intercept* = 0.7, *z* = −2.42, *p* = 0.07	*Intercept* = 0.7, *z* = −2.26, *p* = 0.01

### Discussion

In this experiment, we used two ways to find out if users were aware about the presence of banners. We did not find significant differences in the free recall of banners with difference valence. However, significant differences were found for recognition. Interpreting the data obtained with the regression analysis, we can conclude that banners with neutral valence were recognized better as compared to banners with negative and positive valence.

One of the possible alternative explanations could be that participants spent more time on the neutral banners’ website and that could lead to better neutral banners memorization and recognition. To exclude the influence of amount of time spent on website, we conducted a small additional experiment. Participants had to find the link to three websites with different banners (the same ones were used in the main experiment). Around 24 participants took part in the experiment; the presentation was given within group and counterbalanced with full equalization. The time of the task completion was recorded. Repeated measures ANOVA was used for data analyzes. No significant differences were found, *F* (2,46) = 0.11, *p* = 0.88; *ηp^2^* < 0.01 (see Figure 5).

At that point, participants do notice the banners with neutral valence better as compared to negative and positive ones, but this does not lead to better explicit recall of the contents for the neutral banners. It is assumed that users initially view all the interface’s elements: design, text, menus, and banner ads. Probably, users can notice and even pay attention to banners, but due to the uselessness of information may simply forget it (McCoy et al., 2007). Based on the obtained results, we can say that the valence of the banner can be one of the key factors in classifying information as relevant. Accordingly, the neutral valence indicates the priority of this information. This is likely because marketers often try to make a flashy and catchy advertisement. At the same time, relevant information (useful website elements) is usually presented with neutral valence. This is consistent with the results of the study by Burke et al. (2004), which revealed that animated ads are far less memorable than regular ads.

Thus, according to the results of our research, we can assume that there is so-called active banner avoidance. In other words, users notice banners and are aware of their presence and location. But banners just do not go to a later stage of processing. Such results suggest that the phenomenon of banner blindness is not a special case of blindness due to attentional limitations, but a specific case of irrelevant information ignorance.

The results also demonstrate that banners with positive and negative valence that are close to the task area are recognized better. We believe that this effect may be due to the interaction of two factors. First, previous research has revealed that banners on the right of the website are better remembered by left to right readers (Simola et al., 2011). Also, some studies revealed the attentional capture by emotional stimuli (e.g., Most and Wang, 2011; Kennedy and Most, 2012; Wang et al., 2012). Accordingly, placing emotional banners on the right side increases the probability that they will be detected and remembered (we also address this point more in General discussion).

Banners with neutral valence do not indicate any positive or negative affective information, and therefore are not perceived by users as a typical banner advertisement aimed at grabbing attention. In this regard, users are not inclined to ignore these banners. Thus, to sum it up, we can say that the memorization of banners is influenced by the user experience that speaks about that bright and, probably, emotionally colored images in certain places of the site that contain unwanted information. A sign such as a neutral valence can signal to the user that the information may be useful. But if users still fail to actively ignore banners, emotional ones have a better chance of being remembered. It is also worth noticing that due to the equalization of all groups in terms of arousal, banners with a neutral valence also had a neutral arousal. It is possible that the key factor was not the neutral valence itself, but its interaction with arousal.

## General Discussion

Multiple studies revealed that emotional stimuli attract attention better than neutral stimuli (e.g., Most and Wang, 2011; Kennedy and Most, 2012; Wang et al., 2012). In our study, we got the opposite results: banners with neutral valence were remembered better than negative and positive ones. It can be assumed that when concentrating on task completion, participants ignored negative and positive stimuli. In the study of Pessoa (2005), it was shown that emotional stimuli can be ignored under high load, since without a sufficient amount of attention to the stimulus; information about emotionality will not be processed. In our study, there were similar conditions: participants had a certain task and spent cognitive resources on its completion. Previous research has shown that certain areas of the site are deliberately ignored by users—usually, these are the typical places where advertising is most often located (Benway, 1998; Drèze and Hussherr, 2003). Users initially concentrate on the places where the information relevant to the task is placed and ignored the places that usually contain irrelevant information. For example, horizontal recommending interface layouts lead to bad sales result, because banners usually occupy this space in the screen (Sulikowski, 2020). Thus, usually users are not much distracted by advertising space.

The amount of information on the website was not shown to affect banner blindness (Gorbatova et al., 2020). A point for this can also be found in the evolutionary theory of banner processing. It lies in the fact that ignoring banners is a kind of adaptation that protects users from information overload. First, an inexperienced user notices a banner ad and remembers its basic visual features (e.g., color, shape, and motion). Further, the process of ignoring banners is automatized. Ultimately, users do not notice banners at all. Even animate banners are ignored more often than static (Burke et al., 2005). We can assume that this mechanism works not only with the physical characteristics of banners, but also with the emotional ones. This is how users remember that bright and emotional ads usually contain irrelevant information, which should not be processed, so no cognitive resources are spent on it. In our research, all the banners were located at the same typical site positions: top and side, so users’ experience may lead to banner ignorance (Yu and Tao, 2009). Thus, the users, having a specific goal in the form of finding a link, tended to ignore emotional banners as objects that usually contain irrelevant information.

Based on the ideas about the influence of user experience on the distribution of attention on the digital interface, it can be assumed that users are accustomed to the fact that bright and, probably, emotionally colored ads act as interfering and irrelevant information. We can notice that most of the banner advertisements are bright and colorful, because marketers try to make it conspicuous. According to literature about banner blindness, it could be a mistake. Even Benway (1998) pointed out that increased visibility of a banner worsens its detection, and Ying et al. (2009) confirmed this in their own research, but adding the observation that any method of attracting advertising (animation, brightness, full screen, etc.) only enhances manifestation of the phenomenon of «banner blindness».

We consider it is important to notice that the highest probability of banner detection was observed when the presented banners had both neutral valence and neutral arousal. Perhaps the effect was influenced by the interaction of two factors and not by just neutral valence separately. Probably in this regard, we did not find a significant effect of arousal in Experiment 1. Thus, in future studies, we could test the effect of the interaction of valence and arousal on the manifestation of banner blindness.

However, when considering the recognition of banners in relation to their location, we get opposite results. We have found that banners with a valence other than neutral are better remembered if they are located on the side of the main information of the website. In the previous studies it was shown that banners on the right side of the screen have a higher probability to be noticed (Simola et al., 2011). The closer the advertisement is to the task area, the better it is remembered (Kuisma et al., 2010). This can be explained by the fact that the eyes move from left to right when reading. Accordingly, when reading the end of the lines, attention is drawn to the banner. We believe that the emotionality of the stimulus was important here, as an emotional stimulus could capture attention, as has been shown in previous studies (Beanland et al., 2018). Another explanation is that both neutral and emotional banners capture the attention equally efficient, but positive and negative banners more likely go to the later stages of informational processing, so participants remember these banners better (Buttafuoco et al., 2018).

It would also be of interest to use banners with different levels of valence and arousal on the same webpage in the future studies. According to the evolutionary theory and the present study we can assume that if banners with different valence were located on the same webpage, recognition of the banner with neutral valence would be better. Due to previous user experience, neutral banners would then be categorized as relevant information and emotional ones—as non-relevant, which, in turn, would lead to better processing of the banners with neutral valence.

## Conclusion

Overall, two experiments revealed that the banners with neutral valence are recognized better as compared to the positive and negative ones. Arousal did not seem to affect banner recognition. We showed that processing of banners is largely influenced by the user experience: bright and emotional pictures are typically seen as irrelevant information and ignored by users.

In addition, the highest probability of banner detection was observed for banners that had both neutral valence and neutral arousal, so the effect could be attributed to the interaction of those two factors. Future studies could apply the factorial design to address this issue.

Summing up, we can say that both physical and affective features have major influence on the occurrence of banner blindness. It is likely that when one or more of these characteristics change, it can draw more attention to the image.

## Data Availability Statement

The raw data supporting the conclusions of this article will be made available by the authors, without undue reservation.

## Ethics Statement

Ethical review and approval was not required for the study on human participants in accordance with the local legislation and institutional requirements. The patients/participants provided their written informed consent to participate in this study.

## Author Contributions

EG: originator of the concept, experimental planning, and manuscript preparation. FS: experimental planning, programming, data collection, data analysis, and manuscript preparation. All authors contributed to the article and approved the submitted version.

## Funding

The study was implemented in the framework of the Basic Research Program at the National Research University Higher School of Economics (HSE University) in 2021.

## Conflict of Interest

The authors declare that the research was conducted in the absence of any commercial or financial relationships that could be construed as a potential conflict of interest.

## Publisher’s Note

All claims expressed in this article are solely those of the authors and do not necessarily represent those of their affiliated organizations, or those of the publisher, the editors and the reviewers. Any product that may be evaluated in this article, or claim that may be made by its manufacturer, is not guaranteed or endorsed by the publisher.
